# Depression and Psychological Well-Being as Distinct Constructs: Mutually Exclusive Associations With Biomarker

**DOI:** 10.1093/geroni/igaa057.808

**Published:** 2020-12-16

**Authors:** Kheng Siang Ted Ng, Shu Cheng Wong, Glenn Wong, Ee Heok Kua, Anis Larbi, Roger C M Ho

**Affiliations:** 1 Yong Loo Lin School of Medicine, National University of Singapore, Singapore; 2 Duke-National University of Singapore, Singapore, Singapore; 3 Agency for Science, Technology and Research, Singapore, Singapore, United States; 4 Yong Loo Lin School of Medicine, National University of Singapore, Singapore, Singapore; 5 National University of Singapore, Yong Loo Lin School of Medicine, Singapore, Singapore

## Abstract

Despite increasing emphasis on assessing the mental health of older adults, there has been inconclusive evidence on whether depression and psychological well-being (PWB) are fundamentally distinct constructs or representations of the opposite ends of the mental health spectrum. To instantiate either hypothesis, investigation of the associations between mental health scales and biomarkers have been proposed. First, we assessed depressive symptoms and PWB in community-dwelling older adults (N=59, mean age=67) using the Self-Rating Depression Scale (SDS) and Ryff’s Scale of PWB (comprising six sub-scales). We measured a wide range of immune markers employing ELISA and flow cytometry. Subsequently, we used principal component analysis (PCA) to aggregate and derived biomarker factor scores. Lastly, multiple linear regressions were performed to examine the associations between the scales and the derived biomarker factor scores, controlling for covariates. PCA extracted six biomarker factors. Biomarker factor score 1 was significantly associated with PWB (β=-0.029, p=0.035) and the PWB sub-scale, self-acceptance (β=-0.089, p=0.047), while biomarker factor score 4 was significantly associated with the PWB sub-scale, purpose in life (β=-0.087, p=0.025). On the other hand, biomarker factor 6 was significantly associated with SDS (β=-0.070, p=0.008). There were mutually- exclusive associations between the scales with biomarker factor scores, supporting the hypothesis of distinct constructs. Our findings expanded the biomarkers of depression and PWB, deepening understanding of the biological underpinnings of depressive symptoms and PWB. These findings have implications in field work, since researchers could not infer one construct from the other, the examination of both constructs are essential.

